# Book Reviews

**Published:** 1830-10

**Authors:** 


### BIBLIOGRAPHICAL NOTICES.

*Practical Remarks on the Nature and Effects of the expressed Oil of the Croton Tiglium, with Cases illustrative of its Efficacy in the Cure of various Diseases.* By MICHAEL JOHN SHORT, M.D.—8vo. pp. 63. Longman, London, 1830. Plate.

DR. SHORT's object is to extend the now limited use of the croton oil to diseases in which it has not yet been generally administered, and by the communication of cases which have occurred to him in the course of a long experience of its nature and properties, in India as well as Europe, to raise its reputation in the opinion of the profession. Shortly after Dr. W. E. E. CONWELL, of the Madras service, re-introduced this medicine into European practice, it was highly thought of as a remedy. The adulterations to which it is usually subjected before it comes into the hands of the practitioner are now said to have rendered its use unfrequent in cases where the genuine oil would be very serviceable. An extract is given from a letter of Mr. FROST's, in which he alludes to some of the sophistications to which it has been subjected.

The quickness and certainty of the operation of the genuine croton oil as a purgative, induced Dr. Short to use it in many cases in which it was supposed to be contra-indicated. To remove the erroneous impression that it ought not to be given during inflammatory action, particularly of the primæ viæ or the digestive viscera, two cases of gastro-enteritis are mentioned in which it was administered by Prof. MONCHINI, of Rome, with success. One drop of the oil was mixed with an ounce of simple syrup, and taken at two doses, at half an hour's interval. In each its purgative effects were quick and active.

In a case of acute hepatitis of long duration, Dr. Short employed the croton oil with much advantage. The patient had "innumerable watery stools" after three doses of two drops each, and the operation of the medicine had even produced *deliquium animi.*" The acute form of the disease was thus cut short.

"Much," says Dr. S. "has been urged against the administration of a medicine so active in its operations, in the ordinary forms of disease: I can, however, fully testify to its perfect safety, and its utility in every case where a purgative was indicated, in infancy and adult age, either as a simple purgative, an hydrogogue, or where I desired to produce a sensible effect upon the system, and objected to venesection on account of the permanency of its effects." In corroboration of this opinion letters written by M. Magendie, Dr. Le Fort, and Inspector Tegart, are referred to. The evidence of these gentlemen is strong in favor of the remedy, as an effectual and safe purgative. Dr. Le Fort thinks "it is possibly to be preferred to any other medicine in the greater part of diseases, where it is necessary to open the bowels." Mr. Tegart states that, in the East Indies, "it was found eminently useful as a purgative medicine, exciting the secretions, relaxing the skin, and acting on the whole system as a most efficient febrifuge:" he himself gave it with an "astonishing good effect."

An interesting case of tetanus occurred about three years ago in Mr. Lawrence's practice, in which the croton oil acted very copiously upon the bowels, after various other purgatives had been ineffectually given. Its antispasmodic effects were also very striking in this instance. The patient's recovery was attributed by Mr. L. to the regular exhibition of croton oil and opium. Dr. Short, reasoning from analogy, suggests the use of this remedy in hydrophobia. The hint appears to us well worthy of attention.

Various other cases are adduced by Dr. Short, which tend to prove that the remedy is valuable in many diseases, and that no apprehension need be entertained of the violence of its effects, if it be judiciously employed. He has also applied it externally with much advantage in gout, neuralgia of the facial nerves, &c. Employed in this manner, it produces inflammation and eruption on the part. We believe that the use of the croton oil as an *irritating* liniment originates with Dr. Short: it has often before been used externally to act as a purgative.* He says,

"I am, however, decidedly of opinion, from the experience I have had of its efficacy as a counter-irritant, that it is preferable to all of those now in use: superior to the cantharides in the quickness and certainty of its operation, and in the permanency of its effects; to the ointment of tartarized antimony, for the same reasons, as well as for not producing that excessive pain and constitutional irritation which usually attend the application of the last preparation; and to the common sinapisms, because it stimulates the skin much sooner, diffuses more warmth, and can be better regulated as to the extent of its effects. I have employed it in this way in cases of acute and chronic rheumatism, in gout, in tic douloureux, in glandular and other indolent swellings, and in all cases with the most satisfactory result.

"In cases of pulmonary affection, where it has been desirable to keep up a permanent irritation on the chest, I have found it to answer the purpose infinitely better than either the lyttæ or the tartarized antimony, producing neither the inconvenience nor uneasiness of the one or the other.

* Dict. des Sc. Med. t. vii. p. 412.

"I am of opinion, also, that it is greatly superior to, and more beneficial in its effects than these medicines, from the nature of its operation; as it is pure pus which is secreted from the skin in consequence of its application, which secretion can be extended *ad libitum*, each pustule thus becoming, in some measure, a separate issue; whereas it is merely serum which is discharged when a blister is applied, and there is little discharge at all from the ointment.

"In cases where it is our object to put the system as speedily as possible under the influence of mercurial action, a combination of the oil with the Unguent. Hydrarg. seems to me to answer the purpose peculiarly well. The very stimulating nature of the oil excites the absorbents to extraordinary action; and it is incredible in how short a time the effects of the mercury is manifested from the use of this combination."

We have given the substance of Dr. Short's very interesting remarks upon the utility of this remedy without comment; for we confess that our own experience of it is too limited to justify us in offering any positive opinion upon the subject.

---

*A Treatise on the Minute Anatomy of the Bones.* By ANTONIO SCARPA, Professor of Clinical Surgery in the University of Pavia. Translated from the last Latin Edition.—36mo. pp. 70. London; Bulcock, Chelsea, 1830.

EVERY production from the veteran SCARPA's pen deserves attention. This little work is perhaps more physiologically curious than practically instructive, but still the student will not misspend an hour in perusing it. Scarpa is opposed to the generally received doctrine, which teaches us that the bones are composed of fibres, layers, or tables, placed upon, or so connected and joined with each other, as to have their strata intermingled; and that the strength of the bones depends on the size, number, and length of the layers. He affirms that the greatest part of the whole osseous system is reticular or cellulous. The controversy relative to the minute anatomy of the bones is not whether the structure of the greatest part of the bones is generally cellular or not, but whether the hard and almost rocky walls of the bones, and their compact external crust, no less than their internal substance, partake of this cellular texture. Scarpa has investigated this subject synthetically and analytically. He began by examining the bones in the first rudiments of animation; that is, when the cartilage first changes, and the earliest traces of the future bone begin to appear at the same time. He then deprived the hardest bones of an adult of their earthy particles, and reduced them to their original softness and pellucidness, thinking, as was proved by experiment, that, however entire the maturity of these bones might be, their minute structure would exhibit the same order and relation as was seen in the embryos. HALLER's experiments on the formation of bone in the incubated egg were repeated, and the results are here described. Some rather interesting remarks are also made respecting the various pathological conditions of bones.

